# A minimal biophysical model for the temperature dependence of CO_2_ fixation rates based on macromolecular rate theory

**DOI:** 10.1371/journal.pone.0319324

**Published:** 2025-04-17

**Authors:** Erica J. Prentice, Margaret M. Barbour, Vickery L. Arcus

**Affiliations:** School of Science - Te Aka Mātuatua, University of Waikato, Hamilton, New Zealand; University of Kalyani, INDIA

## Abstract

Accurately predicting how the global environment will change under continued CO_2_ and temperature increases is currently a critical issue. Predictions are dependent on global models that represent this complex system of natural and anthropogenic inputs, responses, and feedback loops. These models must include accurate descriptions of complex biological processes such as photosynthesis, which is currently responsible for the removal of 123 petagrams of atmospheric carbon annually. Here, we develop a simplified approach to model the effect of concurrent changes in temperature and CO_2_ concentrations on the rate of C_3_ carbon fixation. The model simplifies the temperature response of the CO_2_ fixation pathway into a three-parameter curve (as modelled by macromolecular rate theory, MMRT), which incorporates the limitations of RuBisCO kinetics, and CO_2_ and O_2_ solubility as simple system constraints. This framework fully accounts for the temperature and CO_2_ dependence of CO_2_ fixation rates in sweet potato (*Ipomoea batatas*) leaves with just three parameters, in combination with defined biophysical constraints.

## Introduction

Predictions for the trajectory of our global environment are reliant on our ability to accurately model the climate now and into the future under changing temperature and CO_2_ regimes. A central component of global modelling is the ability to predict the rate at which photosynthetic species are removing CO_2_ from the atmosphere, especially extrapolations to changes in CO_2_ fixing capacity in response to increasing temperature and atmospheric CO_2_ concentration. For example, models need to capture the changes in CO_2_ fixing capacity over the predicted future temperature increases of up to four degrees, accompanied by up to a tripling of CO_2_ concentrations by the year 2100 (RCP 8.5) [1].

Rates of net CO_2_ exchange display a curved response to temperature, with increasing rates of CO_2_ fixation up to peak in activity (*T*_opt_), above which rates decline [2–5]. This curvature has been accounted for in various ways. Farquhar and colleagues have fit a Gaussian (which is curved and symmetric about an optimum temperature) to the electron transport component of photosynthesis [6]. In accounting for the temperature response of net CO_2_ assimilation in sweet potato, the limiting processes of carboxylation (*V*_cmax_), electron transport (*J*_max_) and phosphate regeneration are modelled as curves [7]. Medlyn developed a ‘peaked’ model, which incorporates the negative effect of enzyme damage at high temperatures, which is applied to the temperature dependence of *V*_cmax_ and *J*_max_ [4]. Further, the temperature response of net CO_2_ assimilation rates has recently been described in a three parameter single quadratic [2]. Currently, incorporation of CO_2_ fixation at the global scale typically models leaf physiology based on the equations developed by Farquhar and colleagues, parametrising the temperature response of *V*_cmax_ and *J*_max_ based on the fit of modified Arrhenius functions to a range of data [4,8] and incorporating smoothing functions to transition between limiting factors and allow for co-limitation [9–11].

Macromolecular rate theory (MMRT) has been developed to account for the curved temperature dependence of enzyme catalysed rates [12,13]. The basis of MMRT is the expansion of the Eyring-Polanyi equation [14–16] to account for the unusual properties the arise for enzyme catalysis due to the large size of enzymes – hence *macro*molecular rate theory. Specifically, enzymatic reactions undergo a narrowing of the conformational space as the reaction progresses from the enzyme-substrate complex to the tight binding enzyme-transition state complex [17]. As the enzyme-transition state complex has a reduced heat capacity, enzymatic reactions are associated with a negative activation heat capacity ($\Delta C_{p}^{‡}$) [16,17]. The incorporation of a negative $\Delta C_{p}^{‡}$ into the Eyring-Polanyi equation quantifies the degree of negative curvature of enzyme catalysed rates with temperature [14,16]. This accounts for the temperature optimum and decreases in rates at high temperature observed in enzyme catalysis, without the need to invoke enzyme denaturation [12,13].

Given that enzyme catalysed reactions are the underlaying driver of biological rates at increasing scales of complexity, MMRT has been applied to the temperature response of multiple biological systems. This has included the temperature response of rates for both *in vitro* and *in vivo* metabolic pathways [18], as well as various soil processes [15], leaf respiration rates [19] and net ecosystem photosynthesis and respiration [20]. At these increasing scales of biological organisation, the temperature dependence of rates for these various processes are well described by MMRT. The basis for this scaling from enzymes to metabolic pathways has been investigated, showing that the temperature dependent curvature of a metabolic pathway is dependent on the temperature response of the constituent enzymes [18]. This raises the possibility for the application of MMRT to describe the temperature dependence of the CO_2_ fixation as an *in vivo* metabolic pathway. In terms of global scale models of CO_2_ fixation, utilising a curved function like MMRT to describe the temperature response eliminates the need to incorporate smoothing functions between limiting factors [9–11]. Compared to the curved functions which are also used [2], this would concurrently extract information on the thermodynamics of the enzyme driving the process [13,18].

Here, we address this possibility by applying MMRT to describe the temperature response of portions from the CO_2_ fixation pathway of increasing complexity. We find that the temperature response of isolated RuBisCO enzyme, as a large complex macromolecule, is fully accounted for by MMRT. We also find the temperature response of *V*_cmax_ and *J*_max_, representing portions of the CO_2_ fixation pathway measured *in vivo*, are well described by MMRT. We further extend this to apply MMRT to model the temperature response of net CO_2_ fixation rates in the C_3_ species sweet potato (*Ipomoea batatas*) leaves across concurrent changes in temperature and CO_2_ concentration. We find that MMRT, in conjunction with limitations imposed by RuBisCO enzyme kinetics and gas solubility, describes the temperature dependence of net CO_2_ exchange in sweet potato leaves with just three fitted parameters. By incorporating MMRT into this model, the curved temperature response of the enzymes catalysing the CO_2_ fixation process is mechanistically accounted for. This describes net CO_2_ assimilation rates over a 30-degree temperature and 360 ppm_(g)_ CO_2_ range and models the curved response of rates to temperature and associated changes in curvature with altered CO_2_ concentration. Overall, this accounts for the changes in CO_2_ fixation rates due to these two critical environmental factors with a combination of biological and physical parameters in a minimal biophysical model, underpinned by enzyme thermodynamics. For global scale modelling, MMRT presents a promising tool for incorporating a mechanistic understanding to the curvature of the CO_2_ fixation process, while also simplifying input parameters and maintaining model accuracy.

## Model description

We firstly define an artificial state of saturating CO_2_ (substrate), no O_2_ (inhibitor) and ideal light and moisture. The rate of this reaction is defined as:


$$\max imum\;CO_{2}\;assimilation\;rate=v_{\max}=ck_{p}$$ (1)

Where *c* is a constant and *k*_*p*_ is the rate constant for the CO_2_ fixation reaction. At saturating substrate, the reaction rate is not dependent on substrate and in the context of laboratory-based experiments, we assume that the number of CO_2_ fixing centres, c, is constant (over the course of the experiment). We seek to calculate the value for *k*_*p*_ across the temperature range, *k*_*p*_(T). If *k*_*p*_(T) is well defined, then the rates of net CO_2_ exchange in the lab/field may be calculated directly using this function and can be incorporated into models.

We use an existing dataset to define *k*_*p*_(T) as follows: the CO_2_ assimilation rate under experimental conditions is a function of c*k*_*p*_, the concentration of substrate available to RuBisCO (dissolved CO_2_ $\left[CO_{2aq}\right])$ and its observed binding constant ($K_{MCO2}$), the inhibitor concentration (dissolved O_2_ $\left[O_{2aq}\right]$) and its observed binding constant ($K_{iO2}$), and the degree of cooperativity associated with the enzyme catalysed reaction (the hill coefficient, *n*). This is a Michaelis Menten equation with a competitive inhibitor (as in the Farquhar–von Caemmerer–Berry *V*_cmax_ equation [11]) with the addition of a cooperativity term (*n*). These parameters are defined by Equations 2–4. Both $\left[CO_{2aq}\right]$ and $\left[O_{2aq}\right]$ are calculated directly from the partial pressures of these gases at any temperature, simplifying the myriad of complex components related to the bioavailability of CO_2_ into a simple physical constant applicable to C_3_ plants which rely on passive CO_2_ dissolution (Equation 6). Due to this, the model is not applicable to C_4_/CAM plants, as the CO_2_ concentrating mechanisms in these species circumvent the physical constraints of CO_2_ dissolution.


$$CO_{2}\;assimilation\;rate=\frac{v_{\max}{\left[CO_{2\left(aq\right)}\right]}^{n}}{{\left(K_{Mobs}\right)}^{n}+{\left[CO_{2\left(aq\right)}\right]}^{n}}$$ (2)

$$K_{Mobs}=K_{M\;CO_{2}}\left(1+\frac{\left[O_{2\left(aq\right)}\right]}{K_{iO2}}\right)$$ (3)

Combining (2) and (3) gives Equation 4


$$CO_{2}\;assimilation\;rate=\frac{ck_{p}{\left[CO_{2\left(aq\right)}\right]}^{n}}{{\left(K_{MCO2}\left(1+\frac{\left[O_{2\left(aq\right)}\right]}{K_{iO2}}\right)\right)}^{n}+{\left[CO_{2\left(aq\right)}\right]}^{n}}$$ (4)

For sweet potato, Equation 4 was parametrised by a fit of intercellular CO_2_ partial pressure (*C*_i_) vs rate data with high and low O_2_ concentrations to define the response of RuBisCO to substrate and inhibitor (Fig 3) [7]. These data were fit over three temperatures to define the temperature dependence of the parameters. Final model fitting of Equation 1 was achieved with $K_{MCO2}$ independent of temperature (0.094 ppm_(aq)_), whereas $K_{iO2}$ was linearly dependence on temperature, as defined by Equation 5.

**Fig 1:**
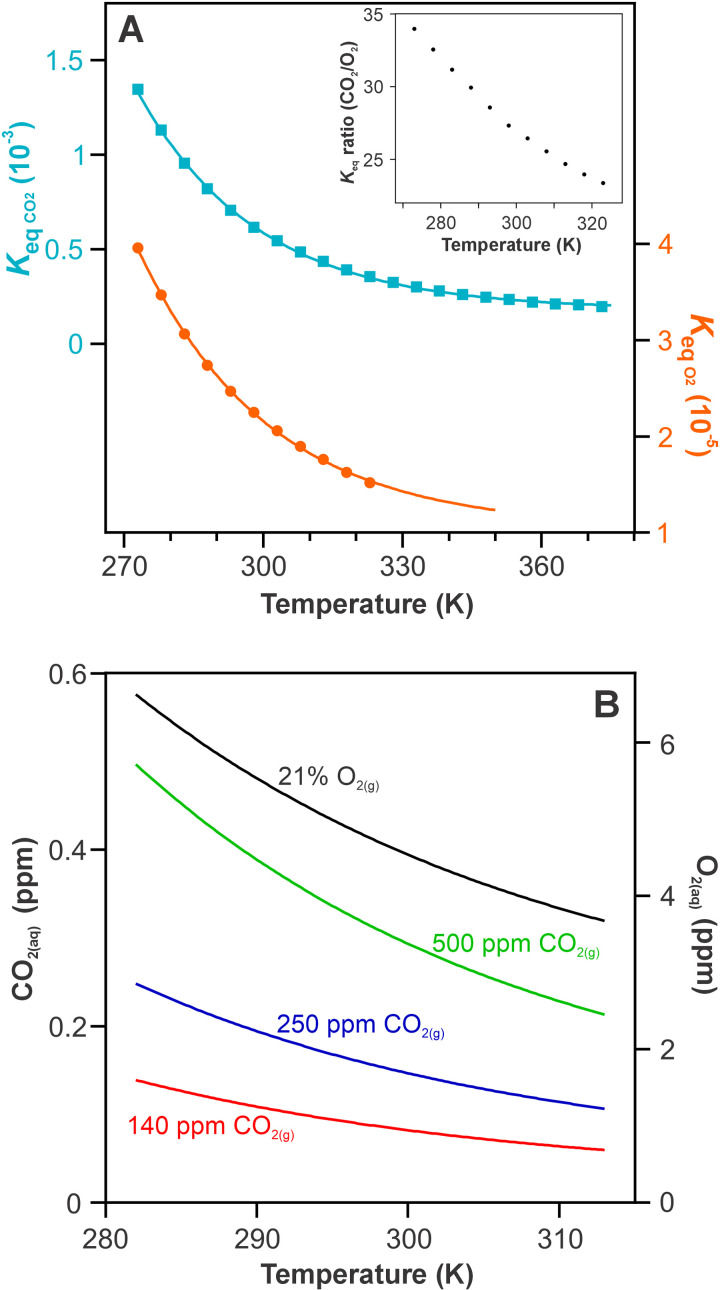
The solubility of CO_2_ and O_2_ in an aqueous system. (A) The change in solubility constants with temperature for CO_2_ and O_2_ (Equation 6). Both gases become less soluble with increasing temperature. (A insert) The ratio of CO_2_ and O_2_ solubility. With increasing temperature, CO_2_ becomes proportionally less soluble compared to O_2_. (B) The effect of temperature on dissolved gas concentrations of current atmospheric O_2_ and three CO_2_ concentrations.

**Fig 2:**
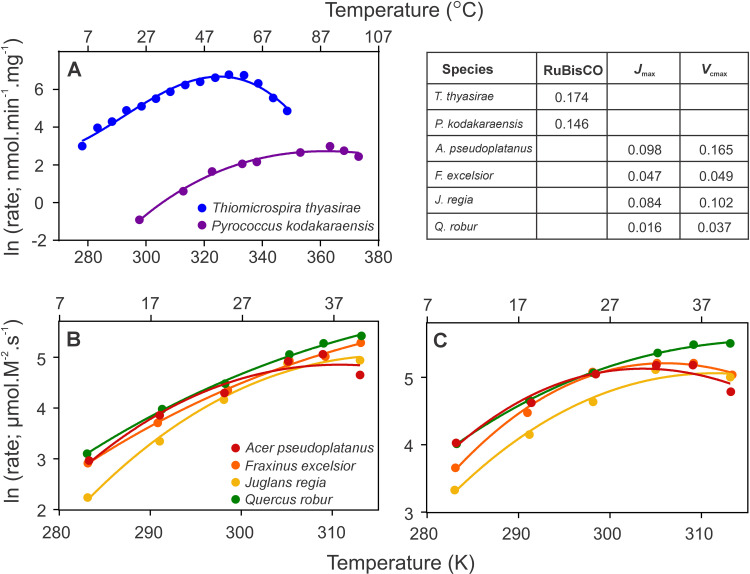
The temperature dependence of rates for isolated RuBisCO (A), *V*_cmax_ (B) and *J*_max_ (C). Rates are fit to MMRT (Equation 7), with the exception of RuBisCO from *T. thyasirae*, which is fit using MMRT with a temperature dependent $\Delta C_{p}^{‡}$ (see S2 supplementary in S1 File). Fitted values are reported in S1 Table in S1 File. RMSE values for the fitting from A-C are given in the table to the top right of the figure.


$$K_{iO2}=\left(-0.483.T\right)+154.6$$ (5)

The data show positive cooperativity, setting an average hill slope of 2.24 over all temperature and O_2_ treatments for the final model. For all data, aqueous CO_2_ and O_2_ concentrations were calculated using the known solubility constants for the specific gases across the temperature range ($K_{eqCO2}$ and $K_{eqO2}$ respectively) [21,22].

The temperature dependence of $K_{eq}$ values are defined by Henry’s law and physical constants for CO_2_ and O_2_ (Equation 6 and Fig 1).


$$lnK_{eq}\left(T\right)=\frac{-\Delta H_{sol}}{R}\left(\frac{1}{T}-\frac{1}{T_{0}}\right)$$ (6)

The maximum rate at a given temperature (*k*_*p*_) is defined by MMRT (Equation 7), where $k_{\text{B}}$ is the Boltzmann constant, *h* is Planck’s constant, *R* is the ideal gas constant, *T* is temperature in Kelvin, $\Delta H_{T0}^{‡}$ and $\Delta S_{T0}^{‡}$ are the activation enthalpy and entropy at the reference temperature (*T*_0_), and $\Delta C_{p}^{‡}$ is the change in heat capacity associated with the reaction. For the analysis here, *T*_0_ was set to 4 °C below the temperature at which the fastest rates were measured (303 K; 30 °C). While the exact value of *T*_0_ does not influence the fitting, this approach of selecting *T*_0_ is consistent with standard MMRT fitting practise.


$$\ln\left(k\right)=\ln\left(\frac{k_{\text{B}}T}{h}\right)-\frac{\left[\Delta H_{T_{0}}^{‡}+\Delta C_{\text{p}}^{‡}\left(T-T_{0}\right)\right]}{RT}+\frac{\left[\Delta S_{T_{0}}^{‡}+\Delta C_{\text{p}}^{‡}\text{ln}\left(\frac{T}{T_{0}}\right)\right]}{R}$$ (7)

Just three parameters are fitted in this equation: $\Delta S_{T0}^{‡}$, $\Delta H_{T0}^{‡}$ and $\Delta C_{\text{p}}^{‡}$. Furthermore, $\Delta S_{T0}^{‡}$ and $\Delta H_{T0}^{‡}$ are linked and define the magnitude of rates. $\Delta C_{\text{p}}^{‡}$ then defines the curvature of the temperature response.

The above model was fit using GraphPad Prism (GraphPad Software, La Jolla, CA, www.graphpad.com; S1 Supplementary in S1 File). Sensitivity analysis of this model was performed by assessing the model fit (*R*^2^) through stepwise alteration of the $K_{MCO2}$, slope of $K_{iO2}$ (Equation 5) and *n* parameters about the typical error range of these values (as given in S4–S9 Tables in S1 File).

## Results

### The temperature response of RuBisCO, *J*_max_ and *V*_cmax_

To test the application of MMRT to CO_2_ fixation, data were fitted for isolated RuBisCO from a bacterial [23] and an archaeal [24] species, as well as *V*_cmax_ and *J*_max_ from a selection of temperate tree species [25] (Fig 2; additional data is in S4 Supplementary section in S1 File). Data for isolated RuBisCO from both species is well described by MMRT (Equation 7) across a wide temperature range (Fig 2A). For the archaeal type III RuBisCO (*Pyrococcus kodakaraensis*), data from 25 to 100 °C is accounted for by the MMRT model (Equation 7). For the bacterial type II RuBisCO (*Thiomicrospira thyasirae*), the fitting requires the inclusion of temperature dependent $\Delta C_{p}^{‡}$, consistent with other high quality, wide temperature range enzyme data (see S2 Supplementary in S1 File for fitting details) [16] . Further, data across a 30 °C range for both *V*_cmax_ and *J*_max_ is well described by MMRT, accounting for the range of curvature in responses observed across the species for both processes.

### The temperature dependence of RuBisCO binding constants in sweet potato

Data for the CO_2_ and O_2_ response of net CO_2_ fixation is available for sweet potato leaves at three temperatures [7]. From this, the binding affinities for the two molecules to RuBisCO can be determined by treating O_2_ as a competitive inhibitor (Equation 4). This reduces the full Farquhar–von Caemmerer–Berry model down to the RuBisCO (*V*_cmax_) portion alone to simplify the analysis for this specific application, while maintaining a full account of the range of *C*_i_ data. The aqueous CO_2_ and O_2_ concentrations are calculated from *C*_i_ based on the equilibrium constant for each gas at the experimental temperature (Equation 6).

**Fig 3:**
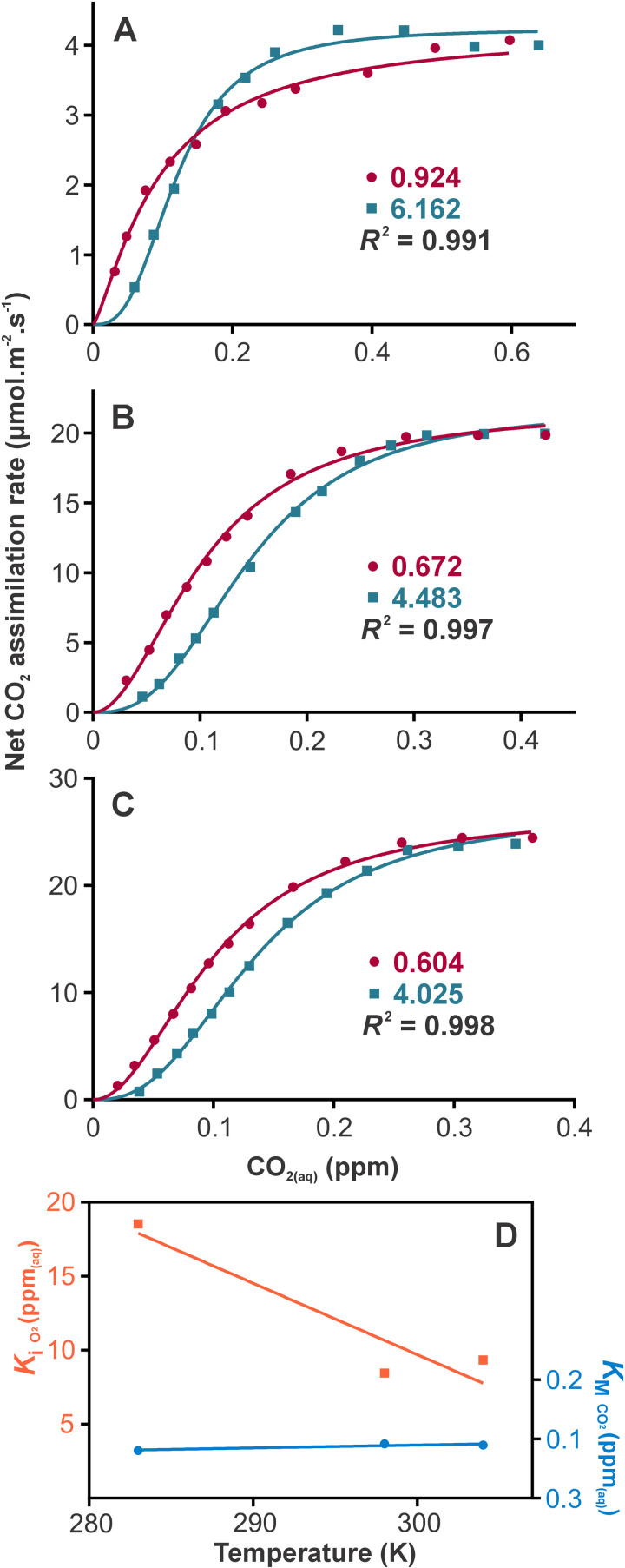
The temperature dependence of RuBisCO binding constants in sweet potato. (A-C) Rate versus aqueous CO_2_ concentration curves for high (200 mbar O_2_(g); red) and low (30 mbar O_2_(g); green) O_2_ concentrations at 10, 25 and 31 °C respectively. Aqueous O_2_ concentrations are given as ppm_(aq)_ within individual graphs. Each temperature is fit with Equation 4, to fit both O_2_ treatments simultaneously to gain binding constants for CO_2_ ($K_{MCO2}$) and O_2_ ($K_{iO2}$). All CO_2_ and O_2_ concentrations are corrected from *C*_i_ values to account for gas solubility at the given temperature. (D) The temperature dependence of binding constants for sweet potato, as determined in fits A-C.

For RuBisCO from sweet potato, affinity for CO_2_ is independent of temperature. The binding constant ($K_{MCO2}$), representing where the RuBisCO pool is half saturated, remains constant at about 0.094 ppm_(aq)_ over a temperature range from 10 to 31 °C (Fig 3). The data also displays positive cooperativity, characterised by an average hill slope of 2.24. In comparison, RuBisCO from sweet potato has two orders of magnitude lower affinity for O_2_ compared to CO_2_, consistent with the biological function of the enzyme. However, the affinity for O_2_ increases with increased temperature (Fig 3D). This increases the relative affinity for O_2_ compared to CO_2_ as temperature is increased.

### The temperature and CO_2_ response of net CO_2_ exchange

Given the parametrised temperature dependence of gas solubilities and RuBisCO binding constants, along with the curved response of enzymatic pathways [18], accounting for the full temperature and CO_2_ dependence of net CO_2_ fixation rates is possible. Temperature data under different *C*_i_ concentrations have previously been collected in sweet potato [7]. These data at three CO_2_ concentrations are simultaneously fit with Equation 1.

**Fig 4:**
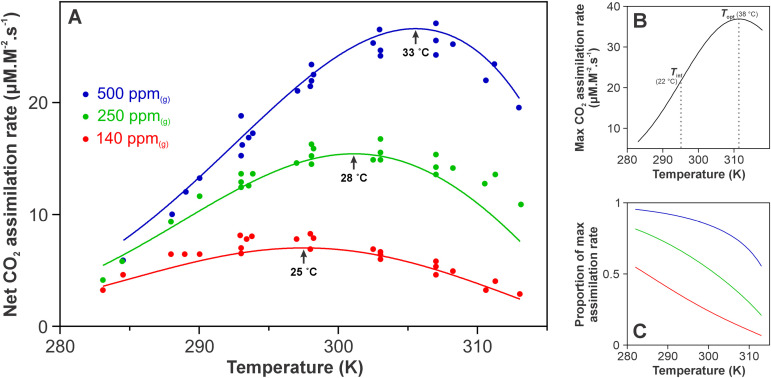
The temperature and CO_2_ dependence of net CO_2_ exchange in sweet potato under optimal light saturation. (A) Global fit of Equation 1 to temperature curves at varying CO_2_ concentrations. The *T*_opt_ under each condition are labelled. *R*^2^ for the global fit =  0.9724. Fitting parameters and further statistics are provided in S3 Table in S1 File. (B) Intrinsic curvature (Equation 7) extrapolated from the fitted data, representing maximum rates of CO_2_ fixation in unlimited conditions (saturating CO_2_, low O_2_, optimal light and moisture). (C) Realised proportion of maximum rates (*k*_p_) given the CO_2_ and O_2_ concentration in solution and binding constants to RuBisCO with temperature. Colouration is the same as panel A for the three CO_2_ concentrations. The deviations away from 100% capacity quantify the physical and biophysical restrictions placed on the CO_2_ fixation system.

Here we find these data can be simply modelled in terms of an intrinsic temperature dependent rate constant *k*_*p*_(T) based on MMRT, the exponential decreases in gas solubility with increases in temperature, and the effects this has on CO_2_ fixation versus photorespiration rates due to $K_{MCO2}$ and $K_{iO2}$ values respectively (Fig 4). The complete dataset, representing an *in vivo* metabolic pathway over a 10–40 °C temperature span, three CO_2_ concentrations and rates varying up to nine fold, can be simultaneously fit with just three parameters (Equation 7), these being the magnitude ($\Delta S_{T0}^{‡}$ and $\Delta H_{T0}^{‡}$) and the curvature ($\Delta C_{p}^{‡}$) of rates.

Sensitivity analysis indicates the model parameters are well defined by the data and are essential for the full fitting of the CO_2_ and temperature response. The ability of the global fit to accurately model the 140 ppm_(g)_ CO_2_ curve is highly sensitive to the input of the $K_{MCO2}$, slope of $K_{iO2}$ and *n*. For example, increasing $K_{MCO2}$ by 10% alters the *R*^2^ for fitting of the 140 ppm_(g)_ CO_2_ curve from 0.76 to 0.41, reducing the predictive power of the model at these low CO_2_ concentrations. Further data for the sensitivity analysis is given in S4–S9 Tables in S1 File.

## Discussion

CO_2_ fixation displays a curved response to changes in temperature, with roughly symmetric decreases in rates either side of an optimum [26]. This curvature is characteristic of a range of biological processes, from enzymes to metabolic pathways, organism growth rates and various ecosystem processes [12,15,18,20]. Across these scales, curvature is well described by MMRT (Equation 7). At the enzyme level, the temperature dependent curvature of rates is a consequence of the large decrease in heat capacity ($\Delta C_{p}^{‡}$) upon progression from the enzyme-substrate complex to the enzyme-transition state. This decrease in heat capacity occurs for the enzyme bound reaction system as the enzyme tightly binds to the transition state species, resulting in a narrowing of the conformational space. A large negative $\Delta C_{p}^{‡}$ defines the temperature dependence of the free energy barrier for a reaction ($\Delta G^{‡}$), expanding the Eyring-Polanyi equation to account for curvature in enzyme rates that is independent of enzyme denaturation [12,13]. Recently, MMRT has been extended to show that the temperature dependent curvature of an *in vitro* metabolic pathway is a function of the curvature of the enzymes contributing to the catalytic cascade [18]. The temperature dependence of leaf [19] and soil [15] respiration, representing *in vivo* metabolic pathways, has also been described by MMRT. Further, analysis of a the global FLUXNET dataset has shown that MMRT can simultaneously describe the temperature dependence of ecosystem photosynthesis and respiration [20]. Considering CO_2_ fixation as an *in vivo* metabolic pathway raises the possibility the process may be described by MMRT, where the temperature response of the pathway is a function of the thermodynamics of the individual enzymes in the cascade. This would then define the inherent temperature response of CO_2_ fixation based on the enzymes catalysing the process, upon which other temperature limitations would layer. Defining the curvature of the photosynthetic pathway with MMRT gives a theoretical basis to the curvature of the temperature response (based on the curvature of individual enzymes), allowing access to information about the thermodynamics of the enzymes underpinning the process. This fitting is mathematically equivalent to the three-parameter single quadratic fit that has previously been used for net CO_2_ assimilation rates [19,27].

To test the applicability of the MMRT equation to the CO_2_ fixation pathway, we fit the temperature response of isolated RuBisCO, as well as *V*_cmax_ and *J*_max_ from a range of sapling species (Fig 2, S1 Fig in S1 File). Across the range of these data representing isolated RuBisCO enzyme and portions of the *in vivo* pathway, MMRT fully accounts for the temperature response of the rates. Further, enzyme data from bacterial type II RuBisCO from *Thiomicrospira thyasirae* requires the extension to include the temperature dependence of $\Delta C_{\text{p}}^{‡}$, a parameter previously applied to high quality enzyme data over wide temperature ranges [16].

We further extended this analysis to develop a minimal model to fully account for net CO_2_ exchange in sweet potato leaves. For this, the response of RuBisCO to changing concentrations of dissolved CO_2_ and O_2_ was defined from existing data (Fig 3). The binding constants of CO_2_ and O_2_ were parametrised with temperature based on an enzymatic competitive inhibition model. The binding constant for CO_2_ ($K_{MCO2}$), is relatively independent of temperature at 0.094 ppm_(aq)_ across this temperature range (Fig 3D), although this binding constant could be expected to increase sharply at higher temperatures [28]. Given a typical CO_2_ concentration and temperature (*C*_i_ of 250 ppm_(g)_ and 18 °C), CO_2_ dissolves to a concentration of 0.19 ppm_(aq)_. Thus, CO_2_ fixation rates are not substrate saturated under typical conditions and are highly sensitive to changes in CO_2_ concentration about current atmospheric levels. The binding constant for O_2_ is two orders of magnitude lower compared to CO_2_, consistent with the biological function of the enzyme. However, the affinity for O_2_ increases with increasing temperature. Due to this, the rate of photorespiration will increase with temperature as the relative binding affinities change.

To be available for fixation, CO_2_ must dissolve from the gas phase in the intercellular space into the aqueous environment of the leaf mesophyll. The movement of CO_2_ from the gas phase to the chloroplast is complex and involves both membrane and cytosolic conductances, many of which are also temperature dependent, variable between species and poorly understood [29–32]. Whilst cognisant of these complexities, for the purposes of this minimal model, these are simplified into a single solubility parameter which captures the broad effects of reduced aqueous CO_2_ with temperature in a well defined physical constant. At all temperatures, the solubility of CO_2_ provides a significant limitation on the substrate available to C_3_ plants for fixation as the equilibrium strongly favours the gas phase (by a factor of 1400 at 20 °C). This equilibrium under a given set of conditions is a physical constant, defined by an equilibrium constant (*K*_eq_). With increasing temperature, CO_2_ solubility decreases exponentially, placing further limits on substrate availability (Fig 1; Equation 6). Incorporating the limitations on CO_2_ supply with temperature via well-defined fundamental restrictions of CO_2_ solubility greatly simplifies the temperature considerations for C_3_ plants, removing reliance on the nuances of membrane and cytosolic conductances.

The rate of net CO_2_ assimilation is complicated by the competing reaction of O_2_ with RuBisCO (photorespiration). Photorespiration temporarily takes a portion of the RuBisCO pool out of CO_2_ fixing capacity, and also requires the release (as CO_2_) of a fixed carbon to recycle the products of two photorespiratory O_2_ turn overs [33]. Net CO_2_ assimilation rates are thus the rate of CO_2_ fixation offset by the rate of CO_2_ release by photorespiration and respiration. The proportion of carboxylation to oxygenation is partially dependent on the aqueous concentrations of CO_2_ and O_2_ (along with the relative binding constants of the two molecules). O_2_ is less soluble than CO_2_, however due to the larger proportion of O_2_ in air, is more prominent in the aqueous phase. For example, under current atmospheric conditions of 210,000 ppm_(g)_ O_2_ (21%) and 400 ppm_(g)_ CO_2_, at 25 °C the gases dissolve to 4.7 and 0.25 ppm_(aq)_ respectively. O_2_ solubility also decreases with increasing temperature, however due to a more gradual decrease in solubility, relative concentrations of dissolved O_2_ compared to CO_2_ increase with temperature (Fig 1A insert). Thus, the physical limitation of gas solubility places further constraints on CO_2_ fixation at elevated temperatures due to the relatively greater solubility of O_2_ compared to CO_2_ and the effects this has on photorespiration rates [26,34–36].

By taking these effects into account, the full response of sweet potato across three different CO_2_ concentrations and a 30°C temperature range is accounted for (Fig 4). Rate decreases from a maximum rate curve (‘intrinsic pathway curvature’, Fig 4B) and shifts in the *T*_opt_ of CO_2_ fixing capacity are captured by the changes in gas solubility (physical constraints), and the effect these have of RuBisCO CO_2_ saturation and photorespiration rates (biological constraints). This maximum rate curve represents the temperature profile of CO_2_ fixation rates in an unconstrained system of saturating CO_2_, vanishingly low O_2_, as well as optimal light and moisture. As CO_2_ concentration is lowered, rates over the whole temperature range drop as RuBisCO becomes less saturated, and photorespiration is higher as the CO_2_:O_2_ ratio is decreased. As CO_2_ is reduced, CO_2_ fixing capacity peaks at a lower temperature due to the lower temperature at which inhibitory ratios of CO_2_:O_2_ are reached when CO_2_ is decreased and O_2_ held constant (Fig 4C). This shifts the *T*_opt_ of CO_2_ fixation from 38 °C to 25 °C between a CO_2_ saturated system (Fig 4B) and 140 ppm_(g)_ CO_2_. The expansion of this analysis to other species is currently limited as this full data set is only available in sweet potato, however the general scale and magnitude of these responses is evident in the literature. A *T*_opt_ shift of similar scale has been reported previously under both high CO_2_ and low O_2_ conditions in *Agropyron smithii* (Western wheat grass) [34], as well as drought stressed *Triticum aestivum* (winter wheat) operating under reduced stomatal conductance and *C*_i_ [37]. Such shifts in the temperature optimum of CO_2_ fixation are critical to accurately capture in models of a climate likely to experience more regular and sever temperatures spikes and increased atmospheric CO_2_ [1].

The capacity of this simple model to incorporate the recent advances in our understanding of the temperature responses of enzymes and metabolic pathways, as well as accounting for the curvature and changes in *T*_opt_ under different CO_2_ regimes has value for global scale modelling. Given the intertwined effects of changing temperature and CO_2_, encompassing the extensive finer details of CO_2_ transport through the mesophyll into a simple solubility term offers great benefit for incorporation into global models, along with other major limiting factors (light, water, nutrient availability). Given the exponential decreases of gas solubility with temperature, this presents a critical component for capturing the subtleties of CO_2_ transport in C_3_ plants into a defined physical constant for understanding large scale CO_2_ fixation over short time scales (in the absence of adaptation mechanisms). To fully realise the potential of this, further data is needed to define the range and variability of the biological parameters of the model (the binding and inhibition constants) across multiple C_3_ species, collet data across a wider temperature range, and assess if biome specific average of these parameters are a possibility.

Currently, terrestrial biosphere models employ a range of approaches to model carboxylation, electron transport and phosphate regeneration rates in response to incident radiation, CO_2_ and temperature (compositions of the major models are summarised in [38]). These models simulate rates based primarily off the equations by Farquhar [11] or Collatz [39]. In addition, these models incorporate a rate-limiting selection between the three processes, and often a smoothing function to even out transitions between the limiting processes and allow for co-limitation [38]. By comparison, the approach presented here fits these limiting processes in one function (Equation 4), simply capturing the net effect of CO_2_ assimilation rates of these three rate-limiting processes and transitions, along with the effects of O_2_. This effectively halves input parameters and removes the need for smoothing functions, reducing model complexity while maintaining output accuracy.

CO_2_ fertilisation has been presented as a mitigating process to rising atmospheric CO_2_ concentrations [40]. The CO_2_ fertilisation effect proposes that elevated CO_2_ concentrations stimulate higher rates of carbon fixation, increasing atmospheric carbon removal to reverse some of the impact of increased anthropogenic inputs. However, when coupled to rising temperatures, this effect will be reduced due to the decreased solubility and bioavailability of the CO_2_ at higher temperatures. For example, from 1991–2015 atmospheric CO_2_ has increased by 40 ppm_(g)_, however the concurrent global average temperature rise of 0.5 °C mitigates the effect of this rise in terms of dissolved CO_2_ bioavailable to plants (per the temperature dependence of CO_2_ solubility; Equation 6). Over this period, no evidence of CO_2_ fertilisation is measurable at a global scale [20]. Future predictions up to the year 2100 indicate that CO_2_ rises (to 700 ppm) are significantly steep compared to projected temperature rises of three degrees to increase dissolved CO_2_ overall from 0.34 to 0.53 ppm_(aq)_ (based on the RCP 6.0 scenario) [41]. This suggests that, in the absence of other major limiting factors [42], increased CO_2_ concentrations will stimulate increased CO_2_ removal. A full understanding of the extent to which CO_2_ fertilisation will mitigate atmospheric CO_2_ increases involves consideration of CO_2_ solubility with temperature, and species-specific data such as RuBisCO binding and inhibition constants to predict wide scale responses to these changes. The analysis presented here provides a simple framework for this modelling, allowing the characterisation of CO_2_ fixation rates of C_3_ plants to concurrent changes in temperature and CO_2_ concentration.

## Conclusions

Here, we show that MMRT can successfully account for the intrinsic curvature of the CO_2_ fixation pathway with temperature. This curvature is well described at the enzyme (RuBisCO), process (*V*_cmax_ and *J*_max_*)*, and full biochemical pathway level. This presents MMRT as a valuable tool for the incorporation into global scale models to account for the temperature response of the enzymes driving the CO_2_ fixation pathway by incorporating fundamental enzyme kinetics. Here, the ‘real world’ limitations of substrate solubility and photorespiration have been successfully incorporated to account for reductions in carbon fixation potential under limited CO_2_ in the C_3_ species sweet potato. Further work is required to assess the range and variability of the parameter set determined here in a wider variety of C_3_ plant species, and how other factors such as light, water and nutrient limitations layer on top of the underlaying enzymatic responses defined here. The model presented here incorporating MMRT along with the limitations imposed by substrate availability and the effects this has on enzyme rates illustrates the potential of this approach in capturing the nuance of temperature-based curvature in a complex metabolic system with a simple model based on fundamental enzyme behaviour. This provides a framework, based on the thermodynamics of enzyme activity, for building other limiting processes of the photosynthetic process onto, as a basis for incorporation into global scale models.

## Supporting information

S1 File Additional information on the methods for model fitting, extended MMRT equation used for enzyme data, fitting parameters for RuBisCO, *V*_cmax_ and *J*_max_ curves, additional fitted thermal curves and fitting parameters, fitting details for net CO_2_ fixation rates, details of the sensitivity analysis, and tables of all data used in this publication. (DOCX)
