# Supplementary material for: A minimal biophysical model for the temperature dependence of CO2 fixation rates based on macromolecular rate theory
Source: PLoS One. 2025 Apr 17;20(4):e0319324. doi: 10.1371/journal.pone.0319324 (PMC12005547; doi:10.1371/journal.pone.0319324)
Supplement: S1 File — (DOCX) [file pone.0319324.s001.docx]

**Supplementary information**

**A minimal biophysical model for the temperature dependence of CO_2_ fixation rates based on Macromolecular Rate Theory**

*Erica J. Prentice^1*^ Margaret M. Barbour^1^ and Vickery L. Arcus*^1*^

^1^School of Science - Te Aka Mātuatua, University of Waikato, Hamilton 3216, New Zealand

For correspondence: [erica.prentice@waikato.ac.nz](mailto:erica.prentice@waikato.ac.nz); [vic.arcus@waikato.ac.nz](mailto:vic.arcus@waikato.ac.nz)

**S1. Methods for model fitting**

All data were fit in GraphPad Prism (GraphPad Software, La Jolla California USA, www.graphpad.com). RuBisCO, *V*_cmax_ and *J*_max_ curves (main text, figure 2) were fit to equation 7 and equation S1.

For net CO_2_ fixation temperature-rate curves, data for all three CO_2_ concentrations were fit simultaneously (equation 1), with a global fit of$\Delta{S_{T}^{\ddagger}}_{0}$, $\Delta{H_{T}^{\ddagger}}_{0}$and $\Delta C_{p}^{\ddagger}$ parameters across the dataset (equation 7), taking into account the effects of changing dissolved CO_2_ and O_2_ concentrations on CO_2_ assimilation rates (equations 2-6).

**S2. The temperature dependence of** $\boldsymbol{\Delta}\boldsymbol{C}_{\mathbf{p}}^{\boldsymbol{\ddagger}}$

The temperature dependence of rates for the RuBisCO enzyme from *T. thyasirae* deviate from equation 1 (main text, figure 2). These data are most simply accounted for by the incorporation of a linearly temperature dependent $\Delta C_{p}^{\ddagger}$. Incorporation of a linearly temperature dependent $\Delta C_{p}^{\ddagger}$ into MMRT gives equation S1. Such behaviour has been documented in high quality, broad temperature data for other enzymes previously [1].

$\ln\left( k \right)=ln\left( \frac{k_{B}T}{h} \right)-\frac{\left[ \Delta H_{T_{0}}^{\ddagger}+\Delta C_{p}^{\ddagger}\left( T-T_{0} \right) \right]}{RT}+\frac{\left[ \Delta S_{T_{0}}^{\ddagger}+\Delta C_{p}^{\ddagger}\ln\left( \frac{T}{T_{0}} \right) \right]}{R}+\frac{m}{2RT}.\left( T-T_{0} \right)^{2}$ (S1)

**S3. Fitting details from figure 1 for RuBisCO, *V*_cmax_ and *J*_max_
Table S1:** **Fitting parameters and details for RuBisCO, *V*_cmax_ and *J*_max_.** figure 1 main text. Δ*H*^ǂ^ and Δ*S*^ǂ^ are quoted at the *T*_0_ value.

| **Species   (process)** | ***T*_0_  (K)** | **Δ*C*^ǂ^_p_  (kJ.mol^-1^.K^-1^)** | **Δ*H*^ǂ^  (kJ.mol^-1^)** | **Δ*S*^ǂ^  (kJ.mol^-1^)** | ***R*^2^** |
| --- | --- | --- | --- | --- | --- |
| ***T. thysirae* *  (RuBicSO)** | 325 | -4.7 ± 0.2  (-0.16 ± 0.03) | 2 ± 4 | 0.20 ± 0.01 | 0.9754 |
| ***P. kodakaraensis*   (RuBisCO)** | 359 | -1.5 ± 0.2 | 1 ± 5 | -0.22 ± 0.01 | 0.9863 |
| ***A. pseudoplatanus*   (*J*_max_)** | 300 | -3.9 ± 0.9 | 11 ±4 | -0.16 ± 0.01 | 0.9510 |
| ***A. pseudoplatanus*   (*V*_cmax_)** | 300 | -4 ± 1 | 37 ± 7 | -0.08 ± 0.02 | 0.9555 |
| ***F. excelsior*   (*J*_max_)** | 300 | -4.2 ± 0.4 | 23 ± 2 | -0.126 ± 0.0006 | 0.9942 |
| ***F. excelsior*   (*V*_cmax_)** | 300 | -1.3 ± 0.4 | 53 ± 2 | -0.032 ± 0.006 | 0.9971 |
| ***J. regia*   (*J*_max_)** | 300 | -3.1 ± 0.8 | 33 ± 4 | -0.09 ± 0.01 | 0.9866 |
| ***J. regia*   (*V*_cmax_)** | 300 | -4 ± 1 | 58 ± 5 | 0.001 ± 0.002 | 0.9920 |
| ***Q. robur*   (*J*_max_)** | 300 | -1.8 ± 0.1 | 30.6 ± 0.7 | -0.100 ± 0.002 | 0.9993 |
| ***Q. robur*   (*V*_cmax_)** | 300 | -1.3 ± 0.3 | 52 ± 2 | -0.0033 ± 0.0005 | 0.9983 |

**For the fitting of T. thysirae, equation S1 is used, including a linearly temperature dependent ΔC^ǂ^_p_. As such, the ΔC^ǂ^_p_ for this fitting is also quoted at T_0_, and the slope for the ΔC^ǂ^_p_ is given in brackets.*

**S4. MMRT fitting of a range of *V*_cmax_ and *J*_max_ temperature curves**

**


Figure S1. The temperature dependence of rates for *V*_cmax_ (A-D) and *J*_max_ (E-H) from a range of species and growth conditions.**
Species and relevant growth conditions are given in the figure. Rates are fit to MMRT (equation 7). Fitted values are reported in Table S2. Data is from (A and E)[2], (B and F)[3], (C)[4], (D)[5], (G and H)[6].

**Table S2: Fitted parameters and statistics for figure S1.** Δ*H*^ǂ^ and Δ*S*^ǂ^ are quoted at the *T*_0_ value.


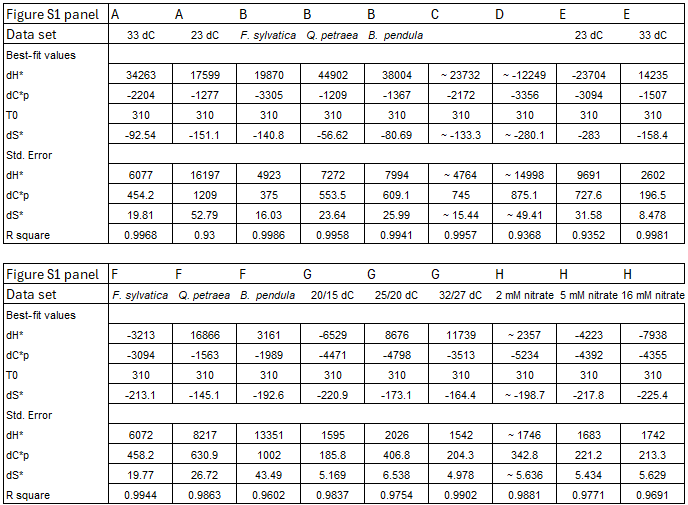


**S5. Fitting details from figure 4 for net CO_2_ exchange rates

Table S3**: Fitting parameters for the net CO_2_ exchange rates from sweet potato leaves, relating to figure 4 in the main text. Δ*C*^ǂ^_p_, Δ*H*^ǂ^ and Δ*S*^ǂ^ are all shared fitting parameters, so are quoted under the global fit. *R*^2^ values are given for the individual curves at each CO_2_ concentration, to show the quality of fit from the global fitting to the individual curves.

| **Curve** | **Δ*C*^ǂ^_p_  (kJ.mol^-1^K^-1^)** | **Δ*H*^ǂ^  (kJ.mol^-1^)** | **Δ*S*^ǂ^  (kJ.mol^-1^)** | ***R*^2^ (adjusted)** | ***Number of data points*** |
| --- | --- | --- | --- | --- | --- |
| ***Global fit*** | -3.0 ± 0.3 | 23 ± 1 | -0.141 ± 0.004 | 0.9724 | 75 |
| ***140 ppm CO_2(g)_*** |  |  |  | 0.7680 | 25 |
| ***250 ppm CO_2(g)_*** |  |  |  | 0.7657 | 25 |
| ***500 ppm CO_2(g)_*** |  |  |  | 0.9578 | 25 |

**S6. Sensitivity analysis details and values**

Sensitivity analysis results are summarised in the following tables. Varied parameters are given in the mint boxes, with the used values under/beside. The initial model parameter is highlighted in grey. *R*^2^ values in the table are coloured from red-yellow-green from low to high *R*^2^. *R*^2^ of the initial model has a bolded box. A dashed line represents model parameters which were unable to fit the data. The initial three tables list the *R*^2^ for the global fit of all three CO_2_ concentration curves, while the following three tables separate the *R*^2^ out per CO_2_ curve from the globally fit model.

**Table S4:** *R*^2^ values for the global fit of all three CO_2_ concentration curves in the sensitivity analysis of *n* and ${{K_{M}}_{CO}}_{2}$ parameters.

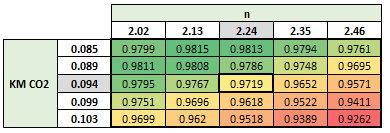


**Table S5:** *R*^2^ values for the global fit of all three CO_2_ concentration curves in the sensitivity analysis of *n* and ${{K_{i}}_{O}}_{2}$ parameters.

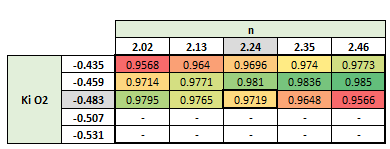


**Table S6:** *R*^2^ values for the global fit of all three CO_2_ concentration curves in the sensitivity analysis of ${{K_{i}}_{O}}_{2}$ and ${{K_{M}}_{CO}}_{2}$ parameters.


**Table S7:** *R*^2^ values for the global fit of the three individual CO_2_ concentration curves in the sensitivity analysis of *n* and ${{K_{M}}_{CO}}_{2}$ parameters.


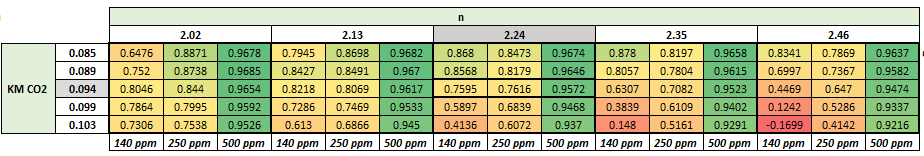


**Table S8:** *R*^2^ values for the global fit of the three individual CO_2_ concentration curves in the sensitivity analysis of *n* and ${{K_{i}}_{O}}_{2}$ parameters.

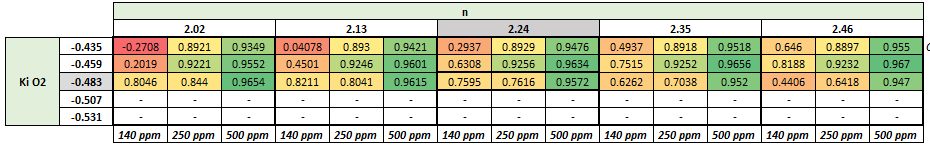


**Table S9:** *R*^2^ values for the global fit of the three individual CO_2_ concentration curves in the sensitivity analysis of ${{K_{i}}_{O}}_{2}$ and ${{K_{M}}_{CO}}_{2}$ parameters.


**S7. Data values used in this publication**

Values given below are as used for this fitting in the stated figures. Original sources for these data are given with the tables.

**Table S10: Data values for Figure 1**

| **T (K)** | **keq CO_2_** | **T (K)** | **keq O_2_** |
| --- | --- | --- | --- |
| 273 | 0.001345 | 273 | 3.96E-05 |
| 278 | 0.00113 | 278 | 3.472E-05 |
| 283 | 0.000955 | 283 | 3.065E-05 |
| 288 | 0.00082 | 288 | 2.739E-05 |
| 293 | 0.000705 | 293 | 2.468E-05 |
| 298 | 0.000615 | 298 | 2.251E-05 |
| 303 | 0.000545 | 303 | 2.061E-05 |
| 308 | 0.000485 | 308 | 1.899E-05 |
| 313 | 0.000435 | 313 | 1.763E-05 |
| 318 | 0.00039 | 318 | 1.627E-05 |
| 323 | 0.000355 | 323 | 1.519E-05 |
| 328 | 0.000325 |  |  |
| 333 | 0.0003 |  |  |
| 338 | 0.00028 |  |  |
| 343 | 0.00026 |  |  |
| 348 | 0.000245 |  |  |
| 353 | 0.000235 |  |  |
| 358 | 0.00022 |  |  |
| 363 | 0.00021 |  |  |
| 368 | 0.000205 |  |  |
| 373 | 0.000195 |  |  |

*Data from [7-11]*

**Table S11: Data values for Figure 2**

| *T. thyasirae* | | *P. kodakaraensis* | |
| --- | --- | --- | --- |
| T (K) | LN(rate: nmol min-1 (mg-1 protein)-1) | T (K) | LN(rate: nmol min-1 (mg-1 protein)-1) |
| 338.60 | 6.32 | 297.70 | -0.91 |
| 333.73 | 6.75 | 312.80 | 0.61 |
| 328.54 | 6.78 | 322.70 | 1.65 |
| 323.78 | 6.62 | 333.10 | 2.06 |
| 318.60 | 6.40 | 338.10 | 2.16 |
| 313.46 | 6.23 | 353.10 | 2.65 |
| 308.34 | 5.88 | 363.30 | 2.99 |
| 303.39 | 5.51 | 368.10 | 2.75 |
| 298.46 | 5.10 | 373.10 | 2.44 |
| 293.25 | 4.89 |  |  |
| 288.30 | 4.30 |  |  |
| 283.31 | 3.96 |  |  |
| 278.05 | 3.00 |  |  |

*Data from [12] and [13]*

**Table S11 continued: Data values for Figure 2**

| *A. pseudoplatanus* | | | *F. excelsior* | |  | *J. regia* | |  | *Q. robur* | |  |
| --- | --- | --- | --- | --- | --- | --- | --- | --- | --- | --- | --- |
| T (K) | Vcmax | Jmax | T (K) | Vcmax | Jmax | T (K) | Vcmax | Jmax | T (K) | Vcmax | Jmax |
| 313.04 | 4.65 | 4.79 | 313.12 | 5.29 | 5.04 | 313.05 | 4.94 | 5.00 | 313.20 | 5.42 | 5.50 |
| 308.96 | 5.06 | 5.18 | 309.24 | 5.02 | 5.21 | 305.08 | 4.88 | 5.11 | 309.03 | 5.28 | 5.48 |
| 305.15 | 4.92 | 5.18 | 305.29 | 4.93 | 5.21 | 298.09 | 4.17 | 4.64 | 305.29 | 5.06 | 5.36 |
| 298.16 | 4.30 | 5.05 | 298.51 | 4.35 | 5.08 | 291.03 | 3.35 | 4.15 | 298.23 | 4.48 | 5.07 |
| 291.03 | 3.86 | 4.62 | 290.82 | 3.71 | 4.48 | 283.13 | 2.24 | 3.33 | 291.32 | 3.98 | 4.66 |
| 283.27 | 2.97 | 4.03 | 283.13 | 2.92 | 3.66 |  |  |  | 283.06 | 3.10 | 4.01 |

*Data from [3]*

**Table S12: Data values for Figure 3**

| **10 degC** |  |  |  |
| --- | --- | --- | --- |
| O2 aq (ppm): | 0.9 |  | 6.2 |
|  |  |  |  |
| CO2 aq (ppm) | Net CO2 assimilation rate (uM.M-2.s-1) | CO2 aq (ppm) | Net CO2 assimilation rate (uM.M-2.s-1) |
| 0.03 | 0.76 | 0.06 | 0.53 |
| 0.05 | 1.26 | 0.09 | 1.29 |
| 0.08 | 1.92 | 0.12 | 1.95 |
| 0.11 | 2.33 | 0.18 | 3.15 |
| 0.15 | 2.58 | 0.22 | 3.54 |
| 0.19 | 3.06 | 0.26 | 3.90 |
| 0.24 | 3.17 | 0.35 | 4.22 |
| 0.29 | 3.38 | 0.45 | 4.21 |
| 0.39 | 3.60 | 0.55 | 3.98 |
| 0.49 | 3.96 | 0.64 | 4.00 |
| 0.60 | 4.07 |  |  |
|  |  |  |  |
|  |  |  |  |

**Table S12 continued: Data values for Figure 3**

| **25 degC** |  |  |  |
| --- | --- | --- | --- |
| O2 aq (ppm): | 0.67 |  | 4.48 |
|  |  |  |  |
| CO2 aq (ppm) | Net CO2 assimilation rate (uM.M-2.s-1) | CO2 aq (ppm) | Net CO2 assimilation rate (uM.M-2.s-1) |
| 0.03 | 2.29 | 0.05 | 1.13 |
| 0.05 | 4.48 | 0.06 | 2.01 |
| 0.07 | 6.96 | 0.08 | 3.86 |
| 0.09 | 8.99 | 0.10 | 5.30 |
| 0.11 | 10.81 | 0.11 | 7.14 |
| 0.12 | 12.59 | 0.15 | 10.42 |
| 0.14 | 14.07 | 0.19 | 14.34 |
| 0.18 | 17.08 | 0.21 | 15.84 |
| 0.23 | 18.69 | 0.25 | 18.03 |
| 0.29 | 19.74 | 0.28 | 19.12 |
| 0.36 | 19.85 | 0.31 | 19.85 |
| 0.42 | 19.87 | 0.37 | 19.94 |
|  |  | 0.42 | 19.96 |

**Table S12 continued: Data values for Figure 3**

| **31 degC** |  |  |  |
| --- | --- | --- | --- |
| O2 aq (ppm): | 0.6 |  | 4.02 |
|  |  |  |  |
| CO2 aq (ppm) | Net CO2 assimilation rate (uM.M-2.s-1) | CO2 aq (ppm) | Net CO2 assimilation rate (uM.M-2.s-1) |
| 0.02 | 1.31 | 0.04 | 0.76 |
| 0.03 | 3.18 | 0.05 | 2.45 |
| 0.05 | 5.57 | 0.07 | 4.34 |
| 0.07 | 8.01 | 0.08 | 6.23 |
| 0.08 | 10.40 | 0.10 | 8.03 |
| 0.10 | 12.73 | 0.11 | 10.03 |
| 0.11 | 14.59 | 0.13 | 12.50 |
| 0.13 | 16.44 | 0.16 | 16.51 |
| 0.17 | 19.86 | 0.19 | 19.29 |
| 0.21 | 22.23 | 0.23 | 21.38 |
| 0.26 | 24.00 | 0.26 | 23.32 |
| 0.31 | 24.45 | 0.30 | 23.66 |
| 0.37 | 24.47 | 0.35 | 23.90 |

*Data from [14]*

**Table S13: Data values for Figure 4**

| 140 ppm CO2(g) |  | 250 ppm CO2(g) |  | 500 ppm CO2(g) |  |
| --- | --- | --- | --- | --- | --- |
| T(K) | Net CO2 assimilation rate (uM.M-2.s-1) | T(K) | Net CO2 assimilation rate (uM.M-2.s-1) | T(K) | Net CO2 assimilation rate (uM.M-2.s-1) |
| 283.1 | 3.2 | 288.0 | 9.4 | 284.5 | 5.9 |
| 284.5 | 4.6 | 290.0 | 11.6 | 288.1 | 10.0 |
| 288.0 | 6.5 | 293.0 | 12.4 | 289.1 | 12.0 |
| 289.0 | 6.5 | 293.6 | 12.6 | 290.0 | 13.2 |
| 290.0 | 6.5 | 293.0 | 12.9 | 293.0 | 15.3 |
| 293.0 | 7.0 | 293.0 | 13.6 | 293.1 | 16.2 |
| 293.0 | 6.5 | 293.9 | 13.6 | 293.6 | 16.9 |
| 292.9 | 8.1 | 297.0 | 14.6 | 293.9 | 17.3 |
| 293.4 | 7.8 | 298.1 | 14.5 | 293.0 | 18.8 |
| 293.8 | 8.1 | 298.1 | 15.2 | 297.1 | 21.1 |
| 297.0 | 7.8 | 298.2 | 15.9 | 298.0 | 21.5 |
| 298.0 | 8.3 | 298.1 | 16.3 | 298.1 | 21.9 |
| 298.2 | 7.9 | 303.0 | 16.8 | 298.2 | 22.5 |
| 298.0 | 6.9 | 303.0 | 15.5 | 298.1 | 23.4 |
| 302.5 | 6.9 | 303.0 | 14.9 | 303.0 | 24.2 |
| 303.0 | 6.7 | 302.5 | 14.9 | 303.0 | 24.7 |
| 303.0 | 6.4 | 307.0 | 15.4 | 302.5 | 25.3 |
| 303.0 | 6.0 | 307.0 | 14.2 | 303.0 | 26.5 |
| 307.0 | 4.6 | 307.0 | 13.6 | 307.0 | 27.1 |
| 307.0 | 5.3 | 308.2 | 14.2 | 307.0 | 25.5 |
| 307.0 | 5.8 | 311.3 | 13.6 | 307.0 | 24.3 |
| 308.2 | 4.9 | 310.5 | 12.8 | 308.2 | 25.2 |
| 310.6 | 3.2 | 313.1 | 10.9 | 311.2 | 23.4 |
| 311.3 | 4.0 | 284.5 | 5.8 | 310.6 | 22.0 |
| 313.1 | 2.9 | 283.1 | 4.1 | 313.0 | 19.5 |

*Data from [14]*

**Table S14: Data values for Figure S1.** Data is labelled per panel labelling in the figure.

| **A** |  | **B** |  | **C** |  | **D** |  | **E** |  | **F** |  |
| --- | --- | --- | --- | --- | --- | --- | --- | --- | --- | --- | --- |
| **T (K)** | **33 dC** | **T (K)** | **F. sylvat** | **T (K)** | **N. acicu** | **T (K)** | **A. alba** | **T (K)** | **23 dC** | **T (K)** | **F. sylvat** |
| 283.09 | 2.57 | 313.26 | 4.90 | 296.92 | 3.56 | 281.17 | -1.13 | 313.16 | 4.47 | 313.35 | 5.05 |
| 292.89 | 3.89 | 305.07 | 4.72 | 300.51 | 3.78 | 281.17 | -0.85 | 308.00 | 4.85 | 305.06 | 5.12 |
| 298.18 | 4.25 | 298.09 | 4.16 | 303.36 | 3.98 | 287.61 | -0.47 | 303.16 | 4.78 | 298.23 | 4.84 |
| 302.87 | 4.61 | 291.17 | 3.49 | 306.58 | 4.10 | 294.02 | 0.21 | 298.31 | 4.61 | 291.19 | 4.32 |
| 308.08 | 5.02 | 283.27 | 2.38 | 309.06 | 4.24 | 294.02 | 0.19 | 293.00 | 4.51 | 283.03 | 3.57 |
| 313.02 | 5.20 |  |  | 311.04 | 4.27 | 293.98 | -0.01 | 283.16 | 3.89 |  |  |
|  |  |  | **Q. petra** |  |  | 298.81 | 0.26 |  |  |  | **Q. petra** |
|  | **23 dC** | 313.20 | 5.42 |  |  | 298.78 | 0.41 |  | **33 dC** | 313.08 | 5.49 |
| 283.00 | 3.15 | 308.96 | 5.22 |  |  | 298.78 | 0.55 | 313.00 | 4.96 | 309.07 | 5.57 |
| 293.15 | 3.69 | 305.08 | 5.00 |  |  | 298.70 | 0.60 | 308.00 | 4.89 | 305.06 | 5.43 |
| 298.01 | 3.98 | 298.02 | 4.30 |  |  | 302.09 | 0.68 | 303.31 | 4.72 | 298.16 | 5.00 |
| 303.13 | 4.35 | 290.89 | 3.83 |  |  | 301.96 | 0.67 | 297.84 | 4.53 | 291.27 | 4.62 |
| 307.99 | 4.68 | 282.92 | 2.86 |  |  | 302.04 | 0.59 | 293.16 | 4.22 | 283.18 | 4.03 |
| 313.19 | 4.41 |  |  |  |  | 301.96 | 0.56 | 283.31 | 3.56 |  |  |
|  |  |  | **B. pend** |  |  | 305.27 | 0.64 |  |  |  | **B. pend** |
|  |  | 313.05 | 5.13 |  |  | 305.18 | 0.47 |  |  | 283.03 | 3.93 |
|  |  | 309.03 | 5.01 |  |  | 305.22 | 0.42 |  |  | 291.19 | 4.33 |
|  |  | 305.08 | 4.88 |  |  | 305.09 | 0.39 |  |  | 298.09 | 4.88 |
|  |  | 298.44 | 4.29 |  |  |  |  |  |  | 305.27 | 5.14 |
|  |  | 291.17 | 3.62 |  |  |  |  |  |  | 313.07 | 5.05 |
|  |  | 283.06 | 2.89 |  |  |  |  |  |  |  |  |

**Table S14 continued: Data values for Figure S1.** Data is labelled per panel labelling in the figure.

| **G** |  |  |  |  |  | **H** |  |  |  |  |  |
| --- | --- | --- | --- | --- | --- | --- | --- | --- | --- | --- | --- |
| **T (K)** | **20/15** | **T (K)** | **25/20** | **T (K)** | **32/27** | **T (K)** | **2 mM** | **T (K)** | **5 mM** | **T (K)** | **16 mM** |
| 288.12 | 4.16 | 295.40 | 4.51 | 289.26 | 3.82 | 296.61 | 3.97 | 289.49 | 3.63 | 288.02 | 4.24 |
| 288.72 | 4.35 | 297.61 | 4.62 | 293.10 | 4.06 | 297.65 | 4.10 | 289.99 | 3.80 | 289.10 | 4.41 |
| 290.87 | 4.42 | 298.47 | 4.71 | 293.76 | 4.29 | 299.14 | 4.17 | 291.39 | 3.94 | 289.21 | 4.46 |
| 291.32 | 4.47 | 299.31 | 4.77 | 296.77 | 4.43 | 299.91 | 4.26 | 293.06 | 4.32 | 290.41 | 4.42 |
| 292.27 | 4.56 | 300.02 | 4.81 | 297.87 | 4.59 | 300.06 | 4.34 | 294.10 | 4.32 | 291.51 | 4.53 |
| 293.10 | 4.64 | 300.95 | 4.88 | 299.84 | 4.74 | 300.74 | 4.33 | 293.95 | 4.24 | 292.49 | 4.61 |
| 293.97 | 4.71 | 301.66 | 4.95 | 301.48 | 4.83 | 301.16 | 4.39 | 295.86 | 4.32 | 293.47 | 4.69 |
| 294.57 | 4.77 | 302.56 | 4.98 | 302.97 | 4.91 | 301.88 | 4.39 | 296.33 | 4.39 | 294.04 | 4.75 |
| 294.57 | 4.83 | 303.60 | 5.06 | 303.12 | 4.94 | 302.32 | 4.43 | 298.48 | 4.43 | 294.45 | 4.80 |
| 295.52 | 4.84 | 304.32 | 5.10 | 303.48 | 4.97 | 303.10 | 4.44 | 297.44 | 4.46 | 295.47 | 4.82 |
| 296.35 | 4.89 | 305.12 | 5.15 | 303.69 | 4.99 | 304.11 | 4.53 | 298.18 | 4.53 | 296.18 | 4.90 |
| 297.34 | 4.97 | 305.48 | 5.21 | 304.49 | 5.02 | 305.51 | 4.57 | 298.92 | 4.58 | 297.04 | 4.96 |
| 298.11 | 5.04 | 306.25 | 5.22 | 305.69 | 5.07 | 307.12 | 4.61 | 299.37 | 4.63 | 297.88 | 5.04 |
| 297.52 | 5.08 | 306.79 | 5.26 | 306.64 | 5.10 | 307.38 | 4.65 | 300.09 | 4.67 | 298.17 | 5.10 |
| 298.56 | 5.13 | 307.74 | 5.30 | 307.65 | 5.14 | 307.92 | 4.66 | 301.19 | 4.69 | 297.10 | 5.16 |
| 299.01 | 5.17 | 308.55 | 5.32 | 308.64 | 5.14 | 308.22 | 4.69 | 301.66 | 4.76 | 298.41 | 5.19 |
| 299.63 | 5.22 | 309.38 | 5.32 | 308.85 | 5.18 | 309.17 | 4.70 | 302.68 | 4.80 | 298.71 | 5.24 |
| 300.32 | 5.27 | 310.19 | 5.37 | 309.50 | 5.17 | 310.09 | 4.69 | 303.33 | 4.83 | 298.97 | 5.26 |
| 300.92 | 5.30 | 311.74 | 5.34 | 310.82 | 5.19 | 310.81 | 4.70 | 303.81 | 4.86 | 299.09 | 5.28 |
| 301.48 | 5.31 | 313.47 | 5.35 | 311.26 | 5.20 | 311.32 | 4.68 | 304.40 | 4.87 | 299.95 | 5.31 |
| 302.20 | 5.36 | 314.69 | 5.31 | 312.13 | 5.19 | 312.15 | 4.69 | 305.06 | 4.89 | 300.85 | 5.35 |
| 303.00 | 5.38 | 315.47 | 5.27 | 312.57 | 5.21 | 312.66 | 4.68 | 305.74 | 4.91 | 301.80 | 5.37 |
| 303.33 | 5.41 | 315.68 | 5.22 | 313.23 | 5.20 | 313.16 | 4.64 | 306.72 | 4.94 | 302.45 | 5.40 |
| 303.72 | 5.43 |  |  | 313.92 | 5.18 | 313.76 | 4.65 | 307.91 | 4.98 | 303.05 | 5.41 |
| 304.55 | 5.45 |  |  | 314.57 | 5.20 |  |  | 308.24 | 4.96 | 303.26 | 5.43 |
| 305.36 | 5.46 |  |  | 315.29 | 5.18 |  |  | 309.20 | 4.95 | 303.70 | 5.45 |
| 306.04 | 5.47 |  |  | 316.63 | 5.18 |  |  | 310.00 | 4.97 | 304.21 | 5.46 |
| 307.12 | 5.47 |  |  |  |  |  |  | 310.86 | 4.96 | 304.96 | 5.46 |
| 307.77 | 5.47 |  |  |  |  |  |  | 311.04 | 4.93 | 305.73 | 5.48 |
| 308.85 | 5.47 |  |  |  |  |  |  | 311.97 | 4.93 | 306.59 | 5.46 |
| 309.09 | 5.46 |  |  |  |  |  |  | 312.62 | 4.91 | 307.87 | 5.46 |
| 309.68 | 5.44 |  |  |  |  |  |  | 313.25 | 4.88 | 308.50 | 5.46 |
| 310.49 | 5.43 |  |  |  |  |  |  | 313.82 | 4.86 | 308.80 | 5.45 |
| 311.47 | 5.42 |  |  |  |  |  |  | 314.65 | 4.88 | 309.36 | 5.44 |
| 312.10 | 5.41 |  |  |  |  |  |  | 315.01 | 4.85 | 310.53 | 5.43 |
| 313.02 | 5.39 |  |  |  |  |  |  | 315.31 | 4.82 | 311.90 | 5.41 |
| 315.17 | 5.38 |  |  |  |  |  |  | 315.93 | 4.80 | 312.49 | 5.37 |
| 315.53 | 5.33 |  |  |  |  |  |  | 317.36 | 4.78 | 313.48 | 5.39 |
| 316.03 | 5.31 |  |  |  |  |  |  |  |  | 313.54 | 5.37 |
| 316.69 | 5.29 |  |  |  |  |  |  |  |  | 314.25 | 5.38 |
|  |  |  |  |  |  |  |  |  |  | 314.91 | 5.37 |
|  |  |  |  |  |  |  |  |  |  | 315.24 | 5.36 |
|  |  |  |  |  |  |  |  |  |  | 315.44 | 5.34 |
|  |  |  |  |  |  |  |  |  |  | 316.07 | 5.35 |
|  |  |  |  |  |  |  |  |  |  | 316.25 | 5.33 |
|  |  |  |  |  |  |  |  |  |  | 316.82 | 5.32 |

*Data sources are given in Figure S1.*

**S8. Supplementary references**

1. Walker EJ, Hamill CJ*, et al.* Cooperative conformational transitions underpin the activation heat capacity in the temperature dependence of enzyme catalysis. *ACS Catalysis*. 2024:4379-94.

2. Benomar L, Moutaoufik MT*, et al.* Thermal acclimation of photosynthetic activity and RuBisCO content in two hybrid poplar clones. *PLoS One*. 2019;14(2):e0206021.

3. Dreyer E, Le Roux X*, et al.* Temperature response of leaf photosynthetic capacity in seedlings from seven temperate tree species. *Tree Physiol*. 2001;21(4):223-32.

4. Miyazawa Y, Otsuki K. Comparison of sapling-level daily light capture and carbon gain between a temperate deciduous and a co-occurring evergreen tree species in the growing season and in winter. *Functional plant biology : FPB*. 2010;37(3):215-22.

5. Robakowski P, Montpied P, Dreyer E. Temperature response of photosynthesis of silver fir (*Abies alba* Mill.) seedlings. *Ann For Sci*. 2002;59(2):163-70.

6. June T, Evans JR, Farquhar GD. A simple new equation for the reversible temperature dependence of photosynthetic electron transport: a study on soybean leaf. *Functional plant biology : FPB*. 2004;31(3):275-83.

7. Carroll JJ, Slupsky JD, Mather AE. The Solubility of Carbon Dioxide in Water at Low Pressure. *J Phys Chem Ref Data*. 1991;20(6):1201-9.

8. Prini RF, Crovetto R. Evaluation of data on solubility of simple apolar gases in light and heavy water at high temperature. *J Phys Chem Ref Data*. 1989;18(3):1231-43.

9. Corovetto R. Evaluation of solubility data of the system CO_2_–H_2_O from 273 K to the critical point of water. *J Phys Chem Ref Data*. 1991;20(3):575-89.

10. Dodds WS, Stutzman LF, Sollami BJ. Carbon dioxide solubility in water. *Ind Eng Chem*. 1956;1(1):92-5.

11. Geng M, Duan Z. Prediction of oxygen solubility in pure water and brines up to high temperatures and pressures. *Geochim Cosmochim Acta*. 2010;74(19):5631-40.

12. Cook CM, Lanaras T*, et al.* Kinetic properties of ribulose bisphosphate carboxylase/oxygenase from *Thiobacillus thyasiris,* the putative symbiont of *Thyasira flexuosa* (Montagu), a bivalve mussel. *J Gen Microbiol*. 1991;137:1491-6.

13. Ezaki S, Maeda N*, et al.* Presence of a structurally novel type ribulose-bisphosphate carboxylase/oxygenase in the hyperthermophilic archaeon, *Pyrococcus kodakaraensis* KOD1. *J Biol Chem*. 1999;274(8):5078-82.

14. Cen Y-P, Sage RF. The regulation of RuBisCO activity in response to variation in temperature and atmospheric CO_2_ partial pressure in sweet potato. *Plant Physiol*. 2005;139(2):979-90.
